# Serum Ferritin Predicts Neither Organ Dysfunction Nor Mortality in Pediatric Sepsis Due to Tropical Infections

**DOI:** 10.3389/fped.2020.607673

**Published:** 2020-12-03

**Authors:** Vijai Williams, Nisha Menon, Prateek Bhatia, Manisha Biswal, Sreejesh Sreedharanunni, Amit Rawat, Muralidharan Jayashree, Karthi Nallasamy

**Affiliations:** ^1^Division of Pediatric Emergency and Intensive Care, Department of Pediatrics, Advanced Pediatrics Centre, Postgraduate Institute of Medical Education & Research, Chandigarh, India; ^2^Division of Pediatric Hematology, Department of Pediatrics, Advanced Pediatrics Centre, Postgraduate Institute of Medical Education & Research, Chandigarh, India; ^3^Department of Medical Microbiology, Postgraduate Institute of Medical Education & Research, Chandigarh, India; ^4^Department of Hematology, Postgraduate Institute of Medical Education & Research, Chandigarh, India; ^5^Division of Pediatric Allergy, Immunology and Rheumatology, Department of Pediatrics, Advanced Pediatrics Centre, Postgraduate Institute of Medical Education & Research, Chandigarh, India

**Keywords:** pediatric, mortality, sepsis, organ dysfunction, tropical infections, scoring, predictor, ferritin

## Abstract

**Objective:** To evaluate serial ferritin levels measured in the initial 72 h of admission as a biomarker for new and progressive multi organ dysfunction syndrome (NPMODS) and mortality (unfavorable outcomes) in critically ill children with sepsis due to tropical infections.

**Material and Methods:** In this prospective observational study from a tertiary care teaching hospital in India, children 3 month to 12 years with a diagnosis of acute febrile illness and any two features suggesting tropical infections [cytopenia (platelet count <1,00,000/cu.mm, total leucocyte count <4,000/cu.mm), hepatomegaly and/or splenomegaly, lymphadenopathy, systemic signs (rash, edema), respiratory distress, and encephalopathy not accounted by localized infection] were eligible for inclusion. Children with known or suspected disorder of iron metabolism were excluded. Primary outcome was to determine the association of serial ferritin levels with mortality and NPMODS. Secondary outcomes included estimation of the prevalence of hyperferritinemia and comparison of risk prediction scores with serial ferritin measurement in predicting unfavorable outcomes.

**Measurements and Main Results:** In the 202 children enrolled, diagnosis could be established in 133 (65.8%) children. Scrub typhus and dengue were the most common infections. Median (IQR) ferritin measured at admission (*n* = 183) and on day 3 (*n* = 120) of hospital stay were 798 (378, 3,205) μg/L and 429 (213,680) μg/L, respectively. Majority (*n* = 180, 89.1%) had MODS at admission defined as per International pediatric sepsis consensus conference. NPMODS occurred in 47 (23.3%) children of whom 37 (18.3%) died. Children with three or less organ dysfunctions had lower mortality. Neither admission ferritin values nor the percentage change over 72 h was different between children with favorable and unfavorable outcomes. Pediatric Risk of Mortality (PRISM-III) and daily Pediatric Logistic Organ Dysfunction score (dPELOD2 score) were significantly different in those with unfavorable outcomes. Admission ferritin levels and percentage change in 72 h had poor discriminatory power for mortality with AUC of 0.53 (0.53, 0.67) and 0.50 (0.50, 0.64), respectively. dPELOD2 had the best discriminatory power for mortality with AUC of 0.89 (0.89, 0.95).

**Conclusions:** Serial ferritin estimation predicted neither organ dysfunction nor mortality in pediatric sepsis with tropical infections. dPELOD-2 and PRISM-III predicted unfavorable outcomes better than ferritin. The current diagnostic criteria for MODS overestimated organ dysfunctions in tropical infections and hence may need modification with further validation in this epidemiological cohort.

## Introduction

Tropical infections are those that are prevalent in, or unique to tropical and subtropical regions. Dengue, malaria, scrub typhus, leptospirosis, and typhoid fever are frequently reported from south-east Asia and India. Some of them occur throughout the year while seasonal predilection is known in infections like dengue and scrub typhus (1). They commonly present as acute undifferentiated fever and sepsis (1–3). Clinical features often overlap especially in presence of organ dysfunction. Laboratory diagnosis largely rely on serology and is time consuming; hence, diagnosis at the outset may be difficult. A syndromic approach utilizing systemic features such as rash, organomegaly or cytopenia is proposed to identify the probable etiology in children presenting with acute febrile illness (4).

Sepsis secondary to tropical infections has notable differences from other bacterial causes. Hematological and/or vascular endothelial involvement are prominent in these infections in association with varying degrees of organ dysfunction. Acute respiratory distress syndrome, encephalitis, myocarditis, acute renal failure and acute hepatic failure are widely reported (3, 5, 6). Organ dysfunction has a close association with mortality which can vary from <5% if single or no organ involvement to >50% with more severe organ dysfunction (7, 8). Multiorgan dysfunction syndrome (MODS) is not uncommon, reported in 17–80% of those requiring intensive care with mortality ranging from 26 to 84% (5, 9, 10). MODS is the result of complex, incompletely understood interplay of pathophysiologic derangements. Prevention of occurrence and progression of organ dysfunction hold promise in reducing mortality. Several predictive and descriptive scoring systems such as Pediatric Risk of Mortality (PRISM III) and daily Pediatric Logistic Organ Dysfunction score (dPELOD2 score) are used to help clinicians recognize organ failures and intervene early. As overall mortality due to sepsis has reduced over past few decades, surrogates like new and progressive MODS (NPMODS) that denote new onset or progressive organ dysfunction during the course are suggested (11).

Several markers are being explored as predictors of unfavorable outcomes in children with sepsis. Ferritin is popular among these. Since the first description by Garcia et al. (12), association between ferritin and poor outcome in children with septic shock has generated great interest among intensivists and clinicians. Studies relating ferritin to various aspects of pediatric sepsis have been published (13–16). Though many centers have now routinely started using ferritin as a marker of severity of illness and in diagnosing infection associated hemophagocytic lymphohistiocytosis (Ia-HLH), there is scarcity of information on this topic in children with tropical infections. Limited evidence in dengue suggest that ferritin may be useful in identifying dengue from other febrile illness as well predicting the severity of illness (17, 18). However, studies exploring serial ferritin levels and its association with NPMODS and mortality in pediatric sepsis, particularly in seasonal tropical infections are not available (11, 19, 20). With this premise, we hypothesized that in children presenting with sepsis due to tropical infections, measuring serial ferritin levels may help identify the severe cases with unfavorable outcomes (NPMODS and 28 days mortality). This data may help in deciding the role of ferritin as a biomarker for predicting sepsis outcome in similar settings.

## Materials and Methods

This was a prospective observational study conducted at the Emergency Department (ED) and Pediatric Intensive Care Unit (PICU) of a tertiary care teaching hospital in North India. The ED caters to children referred from 5 neighboring states with an annual visit of 22,000 and about 10,000 admissions. Our PICU is a 15 bedded unit that admits about 900 children annually. The unit is equipped to provide conventional and oscillatory ventilation, continuous renal replacement, invasive intracranial pressure monitoring and advanced hemodynamic monitoring.

### Study Population

Children aged 3 months to 12 years with acute febrile illness (fever >38.3°C for more than 48 h duration and onset <14 days) without focus with two of the following; cytopenia (platelet count <1 lakhs/cu.mm, total leucocyte count <4,000/cu.mm), hepatomegaly and/or splenomegaly, lymphadenopathy, systemic signs (rash, edema), respiratory distress, and encephalopathy not accounted by localized infection were consecutively enrolled from July 2019 to November 2019, the season with peak incidence of tropical infections. Patients with pre-existing chronic conditions- chronic liver disease, chronic kidney disease, malignancy and/or on immunosuppressive therapy including steroids, autoimmune disorders, familial hemophagocytic lymphohistiocytosis (HLH) and those with disorders of iron metabolism were excluded. Parents/caregivers of patients who met eligibility criteria were approached for possible enrolment in the study. A detailed bilingual patient information sheet was provided to them and a written informed consent was obtained before enrolment. The study began after attaining Institute Ethics Committee approval (NK/5433/Study/594). We included children aged 3 months and older as the iron stores are less influenced by maternal iron stores at this age. The upper limit of 12 years was based on hospital admission policy for Pediatrics.

### Outcome

*Primary outcome* was to determine the association of serial ferritin levels measured in the initial 72 h of admission (day 1 and 3) with mortality and NPMODS (unfavorable outcomes).

*Secondary outcome* included estimation of the prevalence of hyperferritinemia and comparison of risk prediction scores (PRISM III, dPELOD2, and MODS score) with serial ferritin in predicting unfavorable outcomes.

### Definitions

#### Organ Dysfunction

In this study, MODS was defined as ≥2 concurrent organ systems dysfunctions (Supplementary Appendix 1) (21). New MODS was defined as a patient with ≤1 organ dysfunction on day of enrolment and subsequently developed ≥2 concurrent organ dysfunctions. Progressive MODS was defined as a patient with existing MODS (≥2 organ dysfunctions) on day of enrolment who died or developed at least one other concurrent organ dysfunction during the study period. The term NPMODS included patients who had new MODS, progressive MODS or both. MODS or NPMODS was not considered to be present if a single organ dysfunction resolved prior to development of another single organ dysfunction on the next day. Chronic organ dysfunctions established prior to sepsis recognition were not considered to be NPMODS.

#### Scoring Organ Dysfunction

We planned to compare ferritin against predictive and descriptive scores of illness severity for outcome prediction. We used Pediatric Risk of Mortality III (PRISM III) as a predictive score and MODS score and dPELOD-2 scores as descriptive scores. PRISM III is a validated predictive score for mortality in critically ill pediatric patients (22). Most abnormal values within 24 h of admission were used. MODS score is the number of organ dysfunctions (0–6) when using the diagnostic criteria defined according to the international pediatric sepsis consensus guidelines (21). Since MODS score does not differentiate risk of mortality with dysfunctional organ systems differentially, we estimated the dPELOD-2 score that weighs (more points) severity of organ dysfunction and is validated (7).

#### Hyperferritinemia

Hyperferritinemia was defined as ferritin levels more than 500 μg/L (23–25). Since there is currently no established severity grading of hyperferritinemia, we stratified children based on admission ferritin levels into 3 strata. *Stratum 1*: Normal (≤500 μg/L) and with hyperferritinemia (>500 μg/L). *Stratum 2*: Normal (≤500 μg/L), mild (501–3,000 μg/L), moderate (>3,000–10,000 μg/L), and severe (>10,000 μg/L) hyperferritinemia. *Stratum 3*: Normal (≤300 μg/L), mild (301–1,000 μg/L), moderate (>1,000–3,000 μg/L), severe (>3,000–10,000 μg/L), and extreme (>10,000 μg/L) hyperferritinemia. Also, the study group was stratified based on the percentage change in ferritin observed on serial measurements. The degree of ferritin elevation in individual etiological diagnosis was also assessed and compared between survivors and non-survivors.

### Data Collection and Laboratory Investigations

Demographic, clinical and laboratory data were retrieved from medical case records and entered into a pre-designed case record form. Complete blood counts, blood culture, tests for organ dysfunction including renal and liver function were performed at presentation for all cases and were repeated if necessary. Laboratory tests to diagnose the etiology such as Dengue IgM ELISA, NS1 antigen ELISA, PCR, and IgM ELISA for scrub typhus, IgM for leptospirosis, blood smears for malaria, Widal test and serology for hepatitis A and E were carried out based on the clinical syndrome and suspected etiology. We could not perform extended viral diagnostic tests in this study.

Serum ferritin was estimated from the EDTA blood sample collected at admission and on day 3. We chose these time points to identify the prevalence of hyperferritinemia during the early clinical course and to determine its trend irrespective of clinical course. Although more frequent daily serial ferritin measurement would have been helpful, owing to financial limitations, we restricted to two measurements. Plasma was separated after centrifugation at 3,000 rpm for 15 min and stored at −80°C. Samples were processed in batches of 10–15 samples every week using the chemiluminescence principle on the ADVIA Centaur Ferritin System (Siemens Healthcare Diagnostics, Los Angeles, CA) as per manufacturer's instructions.

Treatment related variables, severity score, details of organ supportive therapies, length of PICU stay, and hospital outcome were recorded. dPELOD-2 was done till 5 days or till discharge which ever was earlier. The vasoactive drugs used during therapy were monitored and daily Vasoactive inotrope score (VIS) was calculated (26). Ia-HLH if suspected clinically based on persistence of fever >48 h despite appropriate antimicrobial therapy, unresolving or worsening cytopenia and/or progressive organ dysfunction, necessary work up for the same were done. Since there were limitations in obtaining soluble CD25 levels, decision to treat HLH with immunomodulatory therapy (steroids and/or IvIg) was at the treating clinician's discretion based on the available evidence and clinical course. The study results on ferritin were made available but the investigators were not involved in this decision.

### Statistical Analysis

Descriptive statistics for the demographic data and laboratory parameters were expressed as frequency for categorical variables and as mean [standard deviation (SD)], median [interquartile range (IQR)], for continuous variables depending on normality of distribution. Categorical data were analyzed using chi-square test or Fischer exact test. For two groups comparison, Student *t*-test was used for parametric variables and Mann Whitney test was used for non-parametric variables. Ferritin levels on day 1 and day 3 were compared between survivors and non-survivors and those with NPMODS and without NPMODS. PRISM III, dPELOD2 score (day 1–day 5) and MODS score were compared in those with and without unfavorable outcomes. The area under the receiver operating characteristic (ROC) curve was used to assess sensitivity and specificity to predict unfavorable outcomes.

We performed a sensitivity analysis in those with etiologically confirmed tropical infections after excluding undiagnosed cases. This was done to overcome the bias that could occur due to other bacterial/ viral causes that remained undiagnosed despite best efforts. Similarly, hematological and hepatic dysfunctions are highly prevalent with these etiologies and are likely to recover sooner. This may contribute to a higher organ dysfunction score early in the course as compared to sepsis due to other etiologies. Hence, we also performed a sensitivity analysis restricting to those with high-risk organ dysfunctions defined by any organ dysfunction (cardiac, renal, respiratory or neurological) other than hematological and hepatic involvement. For all tests, a two-sided *P-*value of <0.05 was considered statistically significant. All statistical analyses were performed using SPSS software version 22.0 (SPSS Inc., Chicago, IL).

## Results

### Baseline Characteristics

**Figure 1:**
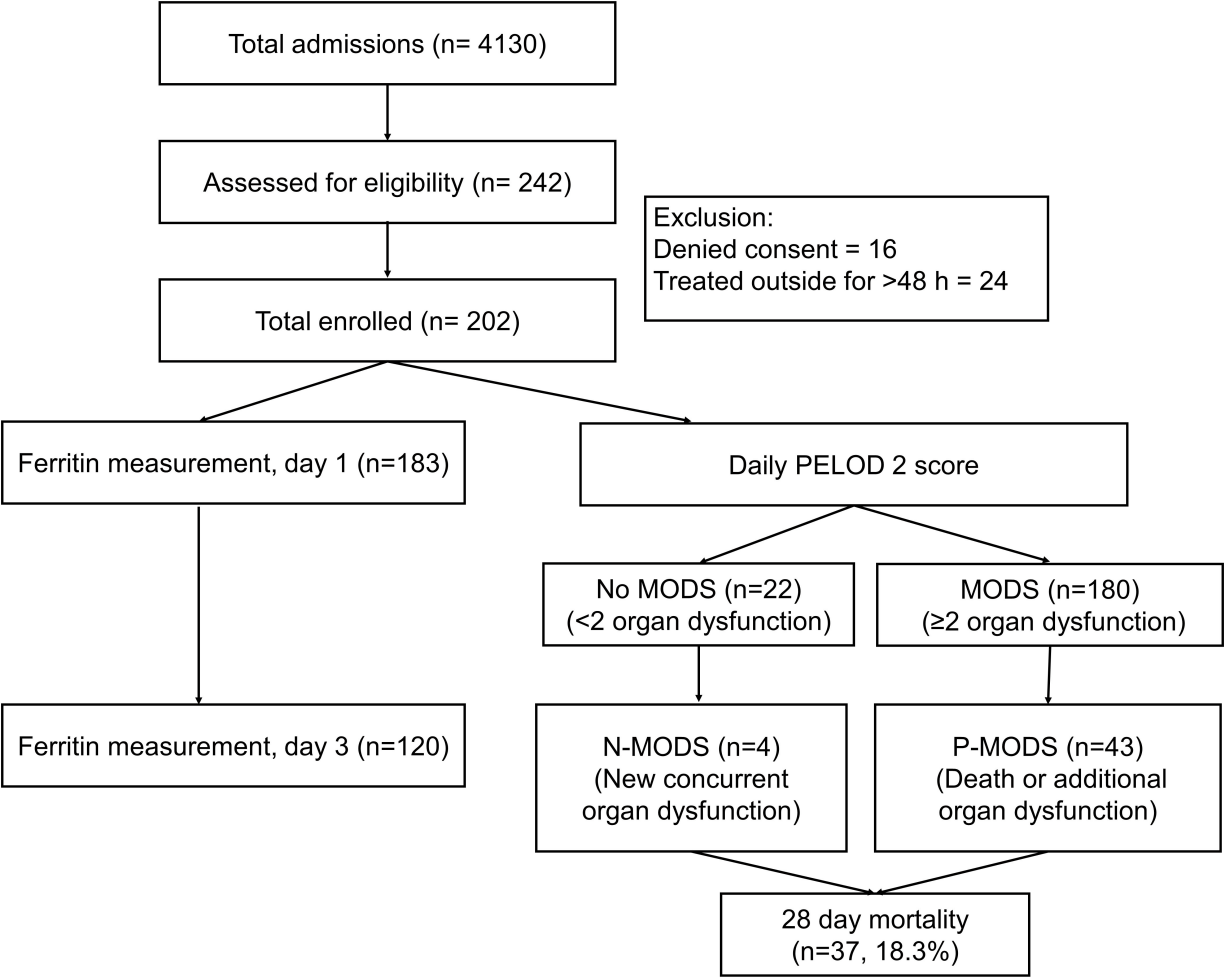
Study flow diagram.

During the study period, 10,158 ED visits and 4,130 admissions were screened. A total of 202 eligible children consented for enrolment (Figure 1). Of these, 183 underwent ferritin measurement on day 1 and 120 children on day 3. The baseline clinical characteristics are shown in Table 1. The median (IQR) age of the study population was 5 (2.5, 8) years, with a male female ratio of 1.2:1. All children had fever at presentation. The common symptoms were edema (*n* = 88, 43.6%) and abdominal pain (*n* = 80, 39.6%). Clinical features like bleeding, altered sensorium and seizures were more common among non-survivors compared to survivors (*p* < 0.05). The median PRISM III score was also higher among non-survivors [15 vs. 7 (*p* < 0.001]. The etiological diagnosis could be established in 133 (65.8%) children (Table 1). Scrub typhus [*n* = 59 (29.2%)] and Dengue [*n* = 41 (20.3%)] were the most common infections diagnosed. Dual seropositivity and/or coinfections were also seen; dengue and scrub typhus in 11 (5.4%) and malaria and scrub typhus in 2 (1%) children. Despite best efforts specific diagnosis could not be ascertained in 34.2% children.

**Table 1:** Baseline clinical characteristics and etiology.

**Parameter**	**Total (*n* = 202)**	**Survivors (*n* = 165)**	**Non-survivors (*n* = 37)**	***P*-value**
Age in years	5 (2.5, 8)	5 (2.5, 8)	3 (2,7)	0.11
Boys, *n* (%)	109 (54)	91 (55.2)	18 (48.6)	0.47
Weight in kg	15 (11.2, 21)	16 (12, 23)	13 (10, 20)	0.06
Weight in Z score	−1.4 (−2.4, −0.6)	−1.4 (−2.3, −0.6)	−1.7 (−2.5, −0.6)	0.25
PRISM III	8 (5, 12)	7 (5, 10)	15 (11, 18)	0.001
Duration of illness in days	5 (4, 7)	6 (4, 7)	5 (3, 8)	0.24
**Clinical features, n (%)**				
Fever	202 (100)	165 (100)	37 (100)	1.0
Edema	88 (43.6)	72 (43.6)	16 (43.2)	0.96
Abdominal pain	80 (39.6)	70 (42.4)	10 (27)	0.08
Fast breathing	46 (22.8)	40 (24.2)	6 (16.2)	0.29
Altered sensorium	47 (23.3)	25 (15.2)	22 (59.5)	0.0001
Seizures	35 (17.3)	21 (12.7)	14 (37.8)	0.0001
Purpuric rash	39 (19.3)	30 (18.2)	9 (24.3)	0.39
Bleeding manifestations	61 (30.2)	45 (27.3)	16 (43.2)	0.05
Oliguria	16 (7.9)	13 (7.9)	3 (8.1)	0.96
Hepatomegaly	155 (76.7)	128 (77.6)	27 (73)	0.55
Splenomegaly	68 (33.7)	55 (33.3)	13 (35.1)	0.83
**Etiology, n (%)**				
Scrub typhus	59 (29.2)	48 (29.1)	11 (29.7)	0.94
Dengue	41 (20.3)	37 (22.4)	4 (10.8)	0.11
Malaria	12 (5.9)	10 (6.1)	2 (5.4)	0.88
Enteric fever	7 (3.5)	6 (3.6)	1 (2.7)	0.78
Japanese encephalitis	1 (0.5)	1 (0.6)	0 (0)	0.63
No specific diagnosis	69 (34.2)	51 (30.9)	18 (48.7)	0.03
**Dual seropositivity and/or coinfections, n (%)**				
Dengue and scrub typhus	11 (5.4)	10 (6.1)	1 (2.7)	0.69
Malaria and scrub typhus	2 (1.0)	2 (1.2)	0 (0)	0.5

*All values expressed as Median (IQR)*.

*PRISM, Pediatric Risk of Mortality*.

### Laboratory Investigations

The median (IQR) hemoglobin was 9.4 (7.7, 11.5) g/dl (Table 2). Thrombocytopenia was seen in about 89% children and median (IQR) platelet count was 42,500 (18,550, 87,750)/mm^3^. Hypoalbuminemia, coagulopathy and elevated lactate were seen more frequently in non-survivors (*p* < 0.0001). Hyperferritinemia was common in study population. Of 183 children who had ferritin estimation, hyperferritinemia >500 μg/L was seen in 124 (67.8%) children. The median ferritin values on day 1 and 3 were 798 (378, 3,205) μg/L and 429 (213, 680) μg/L, respectively. This did not differ significantly among survivors and non-survivors.

**Table 2:** Laboratory investigations of the study population.

**Parameters**	***N***	**Total (*n* = 202)**	**Survivors (*n* = 165)**	**Non-survivors (*n* = 37)**	***P*-value**
Hemoglobin, g/dl	202	9.4 (7.7, 11.5)	9.7 (8.1, 11.7)	7.2 (6.6, 8.7)	0.07
Total leukocyte count, cells/mm^3^	202	11,300 (6,715, 16,107)	10,940 (6,400, 15,900)	11,900 (7,280, 20,100)	0.52
Platelet count, cells/mm^3^	202	42,500 (18,550, 87,750)	42,000 (18,000, 82,000)	44,000 (27,000, 10,900)	0.45
Total bilirubin, mg/dl	127	0.7 (0.4, 1.8)	0.7 (0.4, 1.5)	1.4 (0.6, 3.6)	0.03
Aspartate transaminase (AST), U/L	163	185 (104, 364)	172 (92, 322)	327 (153, 2,187)	0.005
Alanine transaminase (ALT), U/L	160	88 (45, 195)	77 (44, 135)	134 (82, 582)	0.004
International normalized ratio (INR)	137	1.2 (1.0, 1.5)	1.15 (1.0, 1.3)	1.7 (1.3, 2.7)	0.0001
Albumin, g/dl	142	2.4 (2, 2.7)	2.5 (2.2, 2.8)	2.2 (2, 2.7)	0.0001
Creatinine, mg/dl	202	0.4 (0.3, 0.6)	0.4 (0.25, 0.5)	0.5 (0.3, 0.8)	0.001
Lactate, mmol/L	191	2.5 (1.5, 3.8)	2.4 (1.5, 3.4)	3.4 (2.3, 6.0)	0.0001
Severe hypoalbuminemia (<2.5 g/dl), *n* (%)	142	75 (52.8)	56 (48.7)	19 (70.4)	0.04
Coagulopathy (INR>1.5), *n* (%)	139	107 (77)	94 (83.9)	13 (48.1)	0.0001
Elevated ALT, (>2 times IU), *n* (%)	162	40 (24.7)	26 (20)	14 (43.8)	0.005
Day 1 Ferritin, μg/L	183	798 (378, 3,205)	707 (365, 2,937)	1,381 (433, 5,410)	0.39
Day 3 Ferritin, μg/L	120	429 (213, 680)	432 (214, 653)	254 (209, 1,030)	0.99
Percentage change in ferritin	120	44 (19, 70)	46 (19, 72)	44 (21, 67)	0.98

*All values expressed as Median (IQR)*.

### Organ Dysfunction

The most common organ dysfunctions at admission were hematological [*n* = 180 (89.1%)] and hepatic [*n* = 179 (88.6%)] followed by respiratory and renal dysfunctions (Table 3). Eighty-two children (40.6%) were transferred to PICU. Acute respiratory distress syndrome occurred in 77 (38.1%) children. Invasive mechanical ventilation was required for 61 (30.2%) children; 7 required high frequency oscillatory ventilation. Acute kidney injury was noted in 57 (28.2%) children and 20 (9.9%) received renal replacement therapy. The median (IQR) Length of PICU and hospital stay were 4 (3, 7) and 4 (2, 7) days, respectively. Thirty-seven (18%) children died at 28 days follow up.

**Table 3:** Organ dysfunction and therapy details.

**Parameter**	***N***	**Total (*n* = 202)**	**Survivors (*n* = 165)**	**Non-survivors (*n* = 37)**	***P*-value**
**New or progressive MODS**, ***n*** **(%)**	202	47 (23.2)	10 (6.0)	37 (100)	0.0001
N-MODS	22	4 (2.0)	4 (2.4)	0 (0)	0.34
P-MODS	180	43 (21.3)	6 (3.6)	37 (100)	0.0001
**Number of organ dysfunction**					
Day 1	202	2 (2, 3)	2 (2, 4)	4 (3, 4)	0.0001
Day 2	198	2 (2, 3)	2 (1, 3)	4 (4, 5)	0.0001
Day 3	190	2 (2, 3)	2 (1, 3)	4 (3, 5)	0.0001
Day 5	176	1 (1, 1)	1 (0, 2)	5 (4, 5)	0.0001
**PELOD 2 score**					
Day 1	202	3 (2, 6)	2 (2, 3)	8 (7, 10)	0.0001
Day 2	198	2 (2, 5)	2 (2, 3)	9 (6, 10)	0.0001
Day 3	190	2 (1, 4)	2 (1, 2)	9 (7, 10)	0.0001
Day 5	176	1 (0, 2)	2 (1, 2)	8 (7, 12)	0.0001
**Organ dysfunction at admission**, ***n*** **(%)**					
Hematological	202	180 (89.1)	143 (86.7)	37 (100)	0.02
Hepatic	202	179 (88.6)	148 (89.7)	31 (83.8)	0.3
Respiratory	202	63 (31.2)	26 (15.8)	37 (100)	0.0001
Renal	202	57 (28.2)	39 (23.6)	18 (48.6)	0.002
Cardiovascular	202	43 (21.3)	26 (15.8)	17 (45.9)	0.0001
Neurological	202	20 (9.9)	0 (0)	20 (54.1)	0.03
**ARDS**, ***n*** **(%)**	202	77 (38.1)	41 (24.8)	36 (97.3)	0.0001
Invasive mechanical ventilation, *n* (%)	202	61 (30.2)	27 (16.4)	34 (91.9)	0.001
Duration of ventilation, days	61	3 (2, 6)	3 (2, 5)	4 (5, 7)	0.01
HFOV, *n* (%)	61	7 (3.5)	2 (1.2)	5 (13.5)	0.0001
**Shock**, ***n*** **(%)**	202	43 (21.3)	26 (15.8)	17 (45.9)	0.0001
VIS on day 1	202	0 (0)	0 (0)	10 (0, 32)	0.001
VIS on day 3	187	0 (0,10)	0 (0)	25 (13, 81)	0.001
VIS on day 5	175	0 (0)	0 (0)	29 (18, 68)	0.001
**AKI**, ***n*** **(%)**	202	57 (28.2)	39 (23.6)	18 (48.6)	0.002
RRT, *n* (%)	202	20 (9.9)	3 (1.8)	17 (45.9)	0.0001
**Treatment**, ***n*** **(%)**					
Artesunate	202	61 (30.2)	50 (30.3)	11 (29.7)	0.94
Doxycycline	202	175 (86.6)	145 (87.9)	30 (81.1)	0.27
Azithromycin	202	20 (9.9)	14 (8.5)	6 (16.2)	0.33
Immunomodulation (IvIg and/or steroids)	202	14 (6.9)	8 (4.8)	6 (16.2)	0.01
**Outcome**					
Length of PICU stay, days	202	4 (3, 7)	5 (4, 7)	2 (1, 7)	0.001
Length of hospital stay, days	202	4 (2, 7)	5 (3, 8)	2 (2, 4.5)	0.001

*All values expressed as Median (IQR)*.

*PELOD, pediatric logistic organ dysfunction; ARDS, acute respiratory distress syndrome; AKI, acute kidney injury; VIS, Vasoactive inotrope score*.

*HFOV, High frequency oscillatory ventilation; RRT, Renal replacement therapy; IvIg, Intravenous immunoglobulin*.

**Figure 2:**
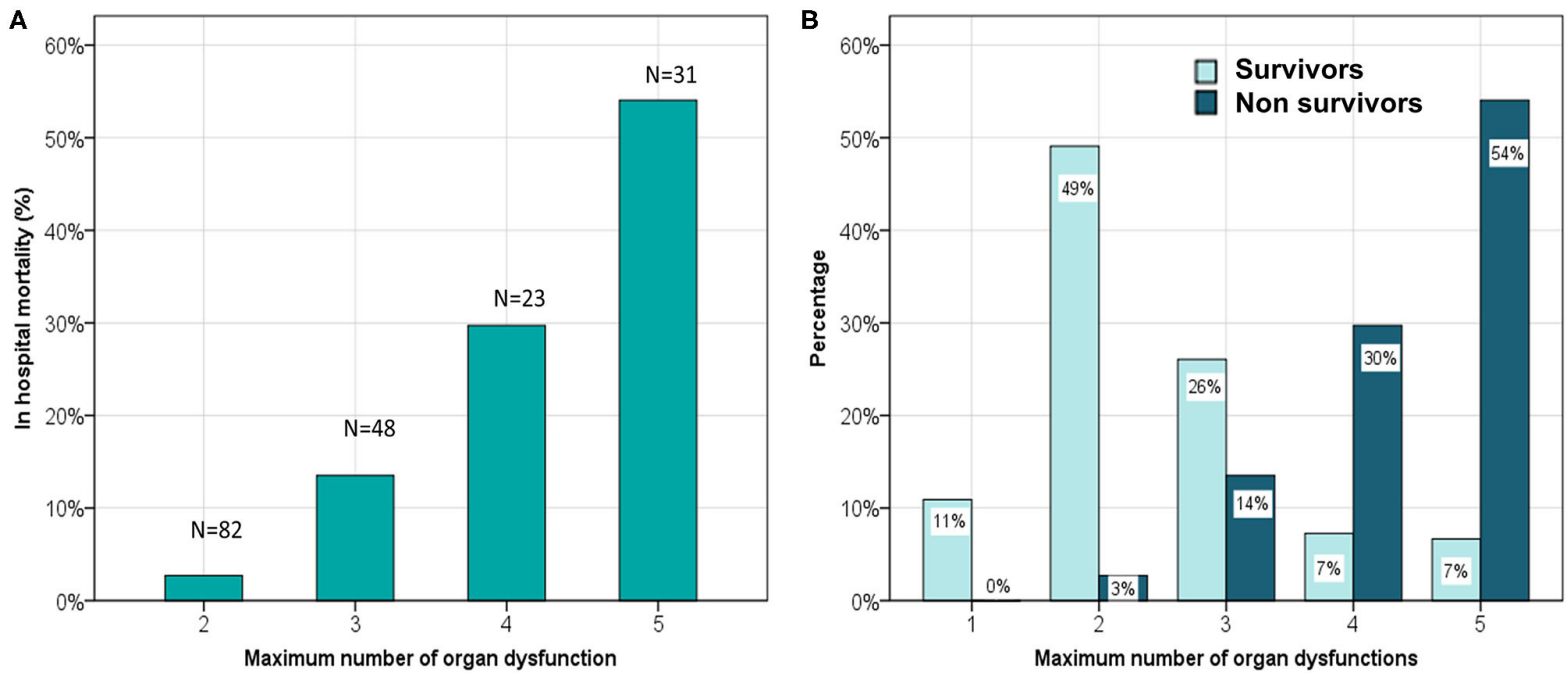
**(A)** Comparison of hospital mortality and the maximum number of concurrent organ system dysfunctions. **(B)** Comparison of maximum number of concurrent organ system dysfunction among survivors and non survivors.

We found that out of 180 children who had MODS at admission, 43 developed progressive MODS. Of 22 children who did not have MODS at admission, 4 developed new MODS. The number of organ dysfunctions at admission were 2 (2,4) and 4 (3,4) among survivors and non-survivors, respectively (*p* < 0.0001). The total number of organ dysfunctions observed during hospital stay was significantly different among survivors and non-survivors; a linear increase in mortality was observed with increase in maximal number of organ dysfunctions (Figure 2). The dPELOD-2 score at admission were 2 (2, 3) and 8 (7, 10) among survivors and non-survivors, respectively (*p* < 0.0001). The score remained significantly higher on all days among non-survivors (*p* < 0.0001).

### Stratification of Ferritin

**Figure 3:**
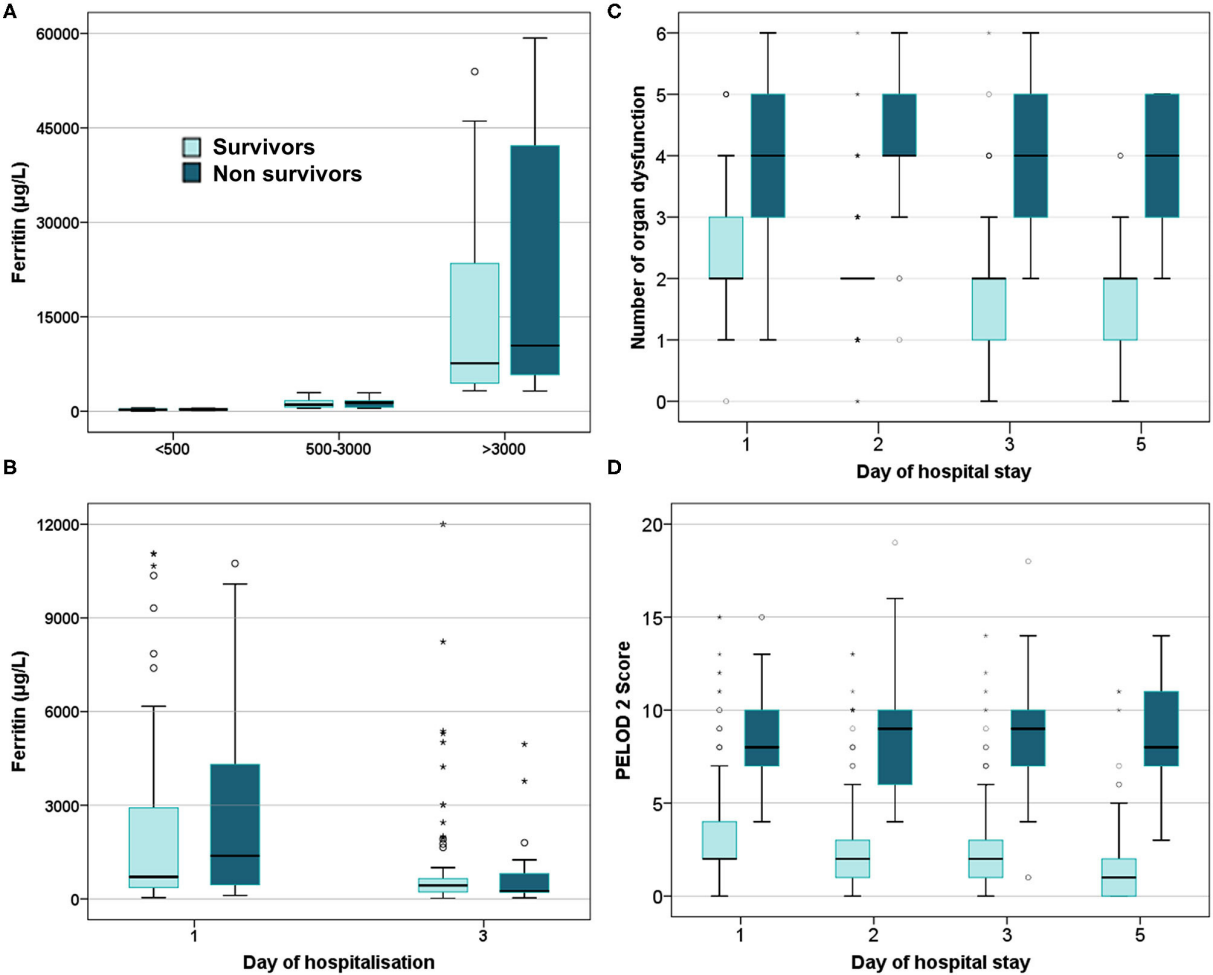
Box plot comparing survivors and non-survivors. **(A)** Ferritin levels at admission; **(B)** Serial ferritin levels measured on day 1 and day 3 of hospitalization; **(C)** Number of organ dysfunction; **(D)** PELOD 2scores.

**Figure 4:**
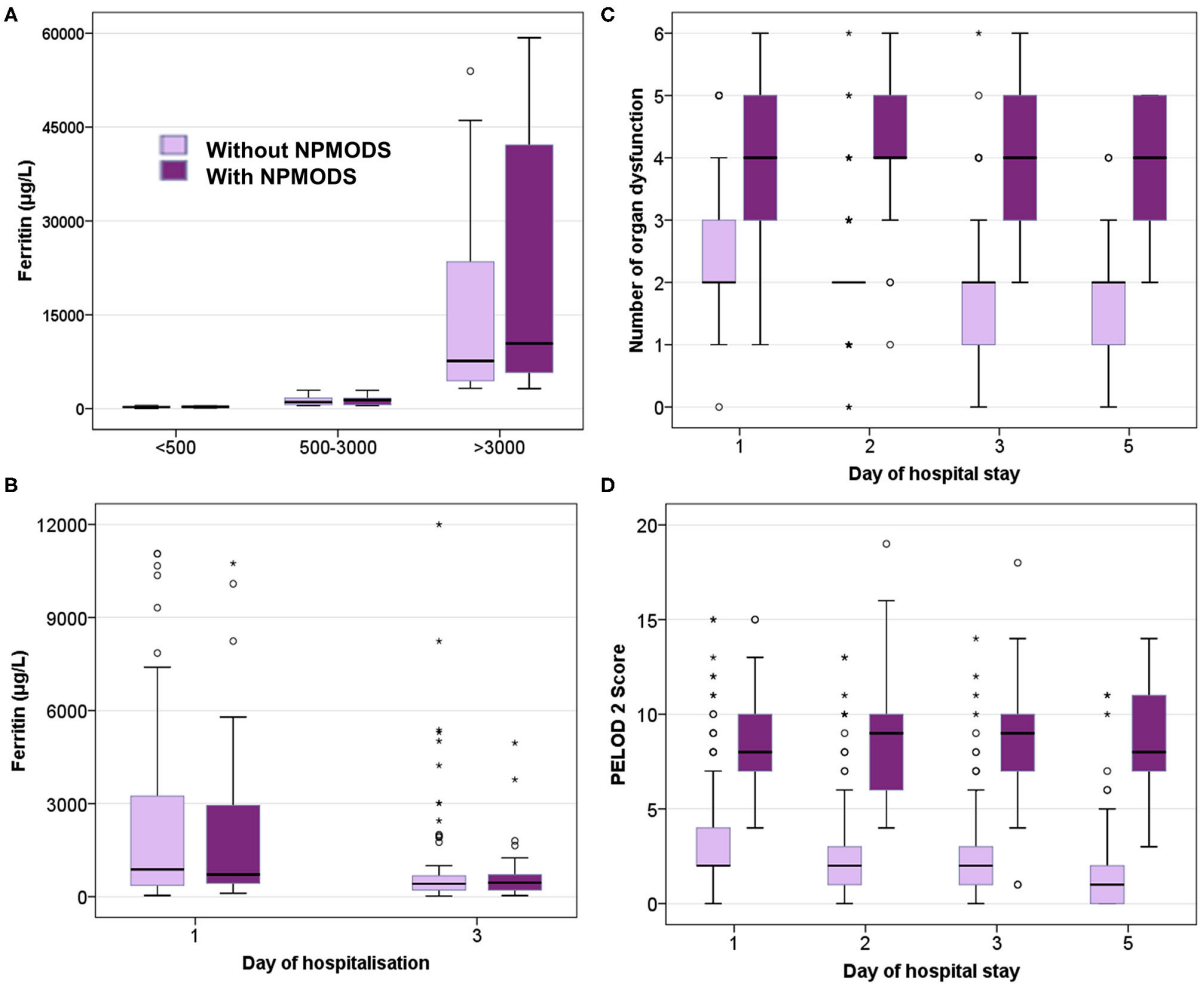
Box plot comparing those with and without NPMODS. **(A)** Ferritin levels at admission; **(B)** Serial ferritin levels measured on day 1 and day 3 of hospitalization; **(C)** Number of organ dysfunction; **(D)**. PELOD 2scores.

On comparing ferritin level among different etiologies, children with dengue showed highest elevation followed by scrub typhus (Table 4). Interestingly, in dengue, survivors had nearly two times higher median ferritin levels as compared to non-survivors. Malaria showed a high median ferritin values among non-survivors (3,514 μg/L vs. 500 μg/L). However, these differences did not reach statistical significance. No significant difference was observed among children across various prespecified strata of hyperferritinemia between survivors and non-survivors (Figure 3) or between those with and without NPMODS (Figure 4). The percentage change in ferritin levels in the initial 72 h also did not differ between the outcome groups. Ferritin levels were not significantly different between survivors and non-survivors when plotted against the number of maximal organ dysfunctions (Supplementary Figure 1).

**Table 4:** Outcome stratified by ferritin levels at admission, percentage change over 48 h and etiology.

**Stratification**	**Total**	**Survivors**	**Non-survivors**	***P*-value**
**Stratification by etiology, Ferritin** **μg/L**				
Dengue	1,913 (621, 5,410)	2,407 (569, 6,173)	1,235 (697, 4,478)	0.82
Scrub typhus	674 (362, 2,892)	665 (360, 2,810)	2,231 (418, 6,514)	0.44
Dengue and scrub typhus	661 (459, 1,514)	669 (378, 1,571)	-	0.38
Malaria	619 (310, 2,131)	500 (232, 1,685)	3,514 (1,212, 4,116)	0.14
Enteric fever	436 (378, 1,457)	557 (416, 1,485)	-	0.13
No specific diagnosis	818 (330, 3,698)	811 (260, 3,356)	1,077 (409, 8,239)	0.23
**Stratification by admission ferritin**, ***n*** **(%)**				
**Stratum 1 (*****n*** **=** **183)**	183	148	35	
≤500	59 (32.2)	49 (33.1)	10 (28.6)	0.61
>500	124 (67.8)	99 (66.9)	25 (71.4)	
**Stratum 2 (*****n*** **=** **183)**	183	148	35	
≤500	59 (32.2)	49 (33.1)	10 (28.6)	0.83
501–3,000	78 (42.7)	63 (42.6)	15 (42.8)	
>3,000	46 (25.1)	36 (24.3)	10 (28.6)	
**Stratum 3 (*****n*** **=** **183)**	183	148	35	
≤300	34 (18.6)	29 (19.6)	5 (14.3)	0.78
301–1,000	62 (33.9)	51 (34.5)	11 (31.4)	
1,001–3,000	41 (22.4)	32 (21.6)	9 (25.7)	
3,001–10,000	24 (13.1)	20 (13.5)	4 (11.4)	
>10,001	22 (12.0)	16 (10.8)	6 (17.2)	
**Stratification by percentage change on serial measurement**, ***n*** **(%)**	120	99	21	
Any percent increase from day 1	17 (14.2)	15 (15.2)	2 (9.6)	0.75
Percent decrease 0–25%	23 (19.2)	18 (18.2)	5 (23.8)	
Percent decrease 26–50%	25 (20.8)	21 (21.2)	4 (19)	
Percent decrease >50%	55 (45.8)	45 (45.4)	10 (47.6)	

*All values expressed as Median (IQR)*.

### Extreme Hyperferritinemia

In this study we found 22 (12%) children with extreme hyperferritinemia. Of them, based on clinician's discretion taking into account of clinical course, organ dysfunction and risk of secondary infections 14 children received immunomodulation with either steroids/immunoglobulins or both. Of them, 6 children died. Soluble CD25 and genetic analysis could not be done in these children. The ferritin levels were not different between survivors and non-survivors in this subgroup.

### Discriminatory Testing by Receiver Operating Characteristics

**Figure 5:**
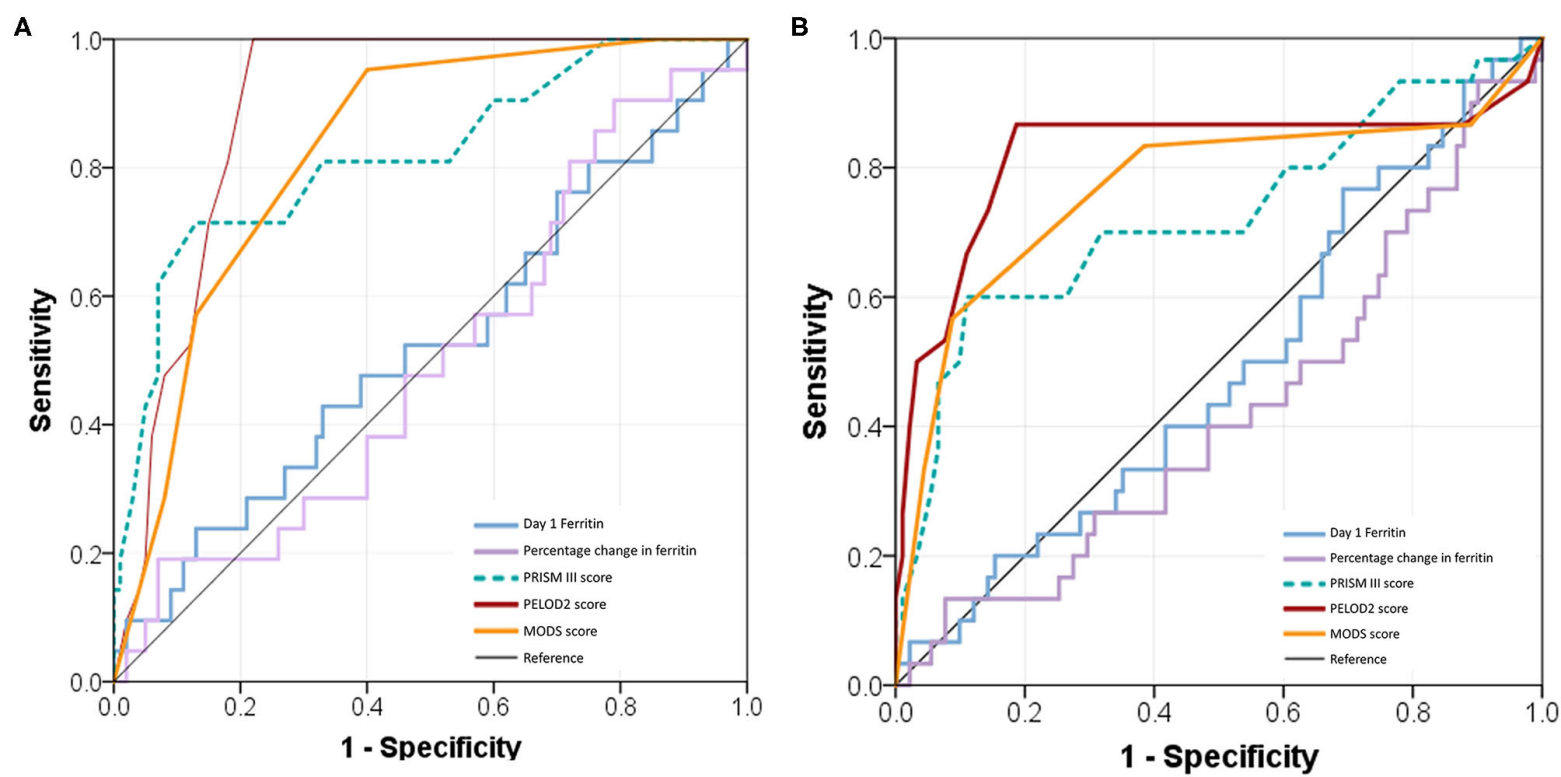
Receiver operating characteristic (ROC) curves comparing admission ferritin, percentage change of ferritin, PRISM III score, PELOD 2 score, and MODS score. **(A)** ROC for predicting 28 days mortality; **(B)** ROC for predicting NPMODS.

Ferritin levels at admission and percentage change in the initial 72 h had poor discriminatory power for mortality with AUC of 0.53 (0.53, 0.67) and 0.50 (0.50, 0.64), respectively (Figure 5A). Admission ferritin >500 μg/L had 62% sensitivity and 36% specificity for mortality whereas higher levels >3,000 μg/L had 24% sensitivity and 79% specificity in predicting mortality. Similar poor discriminatory power was observed for predicting NPMODS as well (Figure 5B). Risk prediction scores had better sensitivity and specificity in predicting mortality and NPMODS than ferritin. dPELOD2 had the best discriminatory power for mortality with AUC of 0.89 (0.89, 0.95) (Supplementary Table 1).

### Sensitivity Analysis

#### Hyperferritinemia in Etiology Proven Cases

Among 133 cases with definitive etiological diagnosis, 115 underwent ferritin estimation on day 1. The median ferritin at admission was 791 (439, 2,876) μg/L and it decreased by a median of 48% (Supplementary Table 2). We did not find any difference in the ferritin levels measured at admission or its percentage change between survivors and non-survivors.

#### Hyperferritinemia in High Risk Organ Dysfunction

Among 108 cases with high risk organ dysfunction, 101 underwent ferritin estimation on day 1. A linear increase in mortality was observed with increase in number of organ dysfunctions. The median ferritin level in this group was 811 (429, 3,546) μg/L with no difference among survivors and non-survivors (Supplementary Table 3).

## Discussion

In this prospective observational study in children hospitalized for sepsis due to tropical infections, we found that neither a single point nor serial ferritin measurement could predict unfavorable outcomes. Serial clinical monitoring for organ dysfunction and use of scores such as dPELOD-2 had better discriminatory ability in predicting the outcome.

Our cohort comprised of children presenting with sepsis and organ failures during monsoon and post monsoon season in a tropical climate. The etiological organisms causing sepsis during this period are significantly different and most often non-bacterial. Majority of these illnesses are vector borne and caused by viruses, protozoa and rickettsia in addition to bacterial causes. This pattern is quite similar to reports from other parts of South and South East Asia where dengue, scrub typhus and enteric fever are endemic. Undifferentiated febrile illness with thrombocytopenia was the most common presentation reported from India (5).

Organ dysfunction at admission is an important factor that determines the clinical course in children with sepsis. Western data suggest that MODS occurs in nearly 20% of children on admission to PICU (27). In a global point prevalence study (SPROUT) that included both high income and low middle-income countries (LMIC) showed that two thirds of children in PICU had MODS at admission (28). In our study we found that 88% children had MODS at admission. Also, the incidence of NPMODS was 23.3% which was higher than the frequency (13–20%) reported in western studies. This high prevalence of MODS could be due to selection bias as we included children with thrombocytopenia which itself accounted for a single organ dysfunction. In addition, hepatic involvement as determined by elevation of ALT was the second most common organ dysfunction observed in 88%. Mortality increased in a stepwise fashion with increasing number of organ dysfunctions similar to those reported by Lin et al. in their large multicentric study in children with pediatric sepsis (11). However, an important difference in our study is that two and three organ involvement had a mortality of 3 and 14%, respectively, a significantly smaller proportion when compared to Lin et al. study. The correlation was better with higher number of organ dysfunctions (>3). This could be due to high frequency of hematological and hepatic dysfunction that are unique to tropical infections that resolve with therapy. This finding suggests that the existing MODS definition fail to discriminate patients in whom the pathogenesis of organ involvement may be different from bacterial sepsis. It reiterates the need to revisit the organ dysfunction scoring, particularly for hematological and hepatic dysfunction in children with tropical infections where a more stringent criteria could be more discriminatory.

Currently, there is no robust evidence on biomarkers that predict severity of inflammation and organ dysfunction in children with sepsis. This is particularly relevant if the etiological agents are predominantly non-bacterial where use of CRP and procalcitonin have their limitations. Ferritin is recently being explored in pediatric sepsis as a biomarker of severity of illness and underlying Ia-HLH. With advanced understanding of its role in inflammation, infection and malignancy, hyperferritinemia as a syndrome is increasingly recognized in critically ill children. Despite the mention in 2004 HLH criteria, elevation of ferritin >500 μg/L could still be non-specific in infections. In our study, hyperferritinemia was seen in 67.8% children reiterating its function as an acute phase reactant to infection. More than half had mild elevation, but severe hyperferritinemia (>10,000 μg/L) was seen in 12% of children. Majority (85.8%) had a decrease in ferritin levels on serial estimation. This may be related to treatment or natural course of the disease.

Illness-appropriate response in ferritin is believed to correlate with favorable outcome. Both high and very low response were shown to be associated with increased mortality in patients with sepsis. Garcia et al. first described an independent association of ferritin and mortality. They observed a mortality of 23 and 58% when ferritin values were <200 μg/L and >500 μg/L, respectively in small retrospective study of 36 children. Interestingly, patients with ferritin between 200 and 500 had only 9% mortality (12). Bennett et al. in their single center retrospective study observed a stepwise increase in mortality risk in hospitalized children with ferritin ≥1,000 μg/L and ≥3,000 μg/L (13). Horvat et al. (29) have found an association between ferritin levels and mortality using lower cutoff points (373 ng/mL), however the authors included all hospitalized children irrespective of sepsis diagnosis. In another recent large retrospective study by Tonial et al. (16) in 312 children from Brazil, a LMIC setting similar to ours a lower cut off of 150 ng/ml was associated with mortality. The authors also showed a 10-fold increase in mortality with 5-fold increase in ferritin. They demonstrated a linear increase in mortality with higher ferritin quartiles. Interestingly, other than mortality and vasoactive drug-free days, other outcomes such as length of PICU and hospital stay, need for vasoactive drugs and mechanical ventilation and number of organ dysfunctions did not differ between ferritin quartiles. However, all these studies have limitations owing to their retrospective design as ferritin estimation was expected to be ordered in association with worsening clinical condition. The possible confounding of transfusion was also not eliminated.

Our findings in this prospective study in children with tropical infections did not concur with previous observations as we found no difference in survival across various strata of ferritin levels. Similarly, we observed no difference in ferritin levels among survivors and non-survivors with respect to etiological causes of sepsis. The median ferritin in dengue was 1,913 (621, 5,410) μg/L, a higher value than those reported with sepsis due to bacterial infections (12, 13). High ferritin levels have been described in other viral illnesses caused by EBV, hepatitis B and C and HIV (30, 31). Simon et al. in their single center prospective study of 75 children demonstrated an association of high ferritin in those with viremia and the elevation was proportionate to the degree of viremia (32). In our cohort, children without a specific diagnosis also had hyperferritinemia; whether this group had an underlying viral etiology remains to be explored.

Bacterial infections require iron for survival. Bacterial infections are usually associated with sequestration of iron and increased ferritin expression both by bacterial antigenic products as well as proinflammatory cytokines as an acute phase marker. However, in viruses, increased ferritin expression at diagnosis has been speculated to be a defense mechanism unless the checkpoints fail and HLH sets in when activating ferritin may harm the host. This hypothesis is more relevant in dengue infection where *in-vitro* studies suggested that ferritin binds to high molecular weight kininogen and hence blocks the release of bradykinin, a potent vasodilator that could contribute to capillary leak syndrome (33). Hence, ferritin elevation in dengue infection is possibly a host protective defense mechanism and likely explanation for the higher median ferritin at diagnosis in dengue survivors. In a study by van de Weg et al. (17) hyperferritinemia was significantly higher in dengue cases than other febrile illnesses and authors noted an odds ratio of 6 with specificity of 88% for high median ferritin to predict dengue infection. Moreover, in their study hyperferritinemia at various time points was associated with high viremia.

Ferritin showed poor discrimination for the occurrence of NPMODS and mortality. Higher dPELOD score was seen in NPMODS and non-survivors suggesting it as a good tool in predicting unfavorable outcome. PRISM III also performed better than serial ferritin. We feel that clinical and laboratory monitoring of organ function along with the use of available predictive and descriptive scores would be more beneficial than routine ferritin estimation in children with tropical infections.

## Strengths and Limitations

Our study has some important strengths. This is one of largest prospective pediatric studies on sepsis due to tropical infections. This is the first instance where serial ferritin measurements and its percentage change is being explored as a marker of unfavorable outcome in a large cohort of children with sepsis. We had a robust follow up till 28 days with daily evaluation of organ dysfunction. However, a few limitations need mention. Our study is primarily limited to tropical infections and hence has limited generalizability in sepsis due to other bacterial causes. A specific etiology could not be ascertained in a third of enrolled children, however, this proportion is similar to previous reports from similar settings (3). Since extensive evaluation for viral etiologies like Epstein Barr virus and cytomegalovirus could not be done in this cohort, viremia causing hyperferritinemia remains unexplored. We could not assess or combine other biomarkers such as CRP or procalcitonin in this analysis. The prevalence of iron deficiency could not be quantified in this study as markers for iron status was not done.

## Conclusion

Serial ferritin estimation neither predicts organ dysfunction nor mortality in pediatric sepsis with tropical infections. dPELOD-2 and PRISM III scores predicted unfavorable outcomes better than ferritin. The current diagnostic criteria for MODS are useful however, they tend to overestimate organ dysfunctions in tropical infections. Hence, there is an urgent need for modification and validation in this epidemiological cohort.

## Data Availability Statement

The raw data supporting the conclusions of this article will be made available by the authors, on reasonable request.

## Ethics Statement

The studies involving human participants were reviewed and approved by Institute Ethics Committee, Postgraduate Institute of Medical Education & Research, Chandigarh, India (NK/5433/Study/594). Written informed consent to participate in this study was provided by the participants' legal guardian/next of kin.

## Author Contributions

KN had full access to all the data in the study and took responsibility for the integrity of the data and the accuracy of the data analysis. All authors participated in the study concept and design. VW and NM had collected, analyzed, and interpreted study data. VW, NM, and KN drafted the manuscript. PB helped in ferritin estimation. MB helped in laboratory confirmation of scrub typhus through PCR and ELISA. SS did bone marrow examination in those suspected with HLH. MJ and SS gave critical inputs during revision of the manuscript. MJ and KN provided administrative, technical, or material support. All authors read and approved the final manuscript.

## Conflict of Interest

The authors declare that the research was conducted in the absence of any commercial or financial relationships that could be construed as a potential conflict of interest.

## Supplementary Material

Supplementary Figure 1 Box plot comparing ferritin with degree of organ dysfunction and survival.

## Abbreviations

AKI: acute kidney injury
ARDS: acute respiratory distress syndrome
DIC: disseminated intravascular coagulation
ELISA: enzyme linked immunosorbent assay
IVIg: intravenous immunoglobulin
KDIGO: kidney diseases improving global outcomes
MODS: multiorgan dysfunction syndrome
PCR: polymerase chain reaction
PELOD: pediatric logistic organ dysfunction
PICU: pediatric intensive care unit
PRISM: pediatric risk of mortality
VIS: vasoactive inotrope score.
