# Supplementary figures and images for: Serum Ferritin Predicts Neither Organ Dysfunction Nor Mortality in Pediatric Sepsis Due to Tropical Infections
Source: Front Pediatr. 2020 Dec 3;8:607673. doi: 10.3389/fped.2020.607673 (PMC7747694; doi:10.3389/fped.2020.607673)

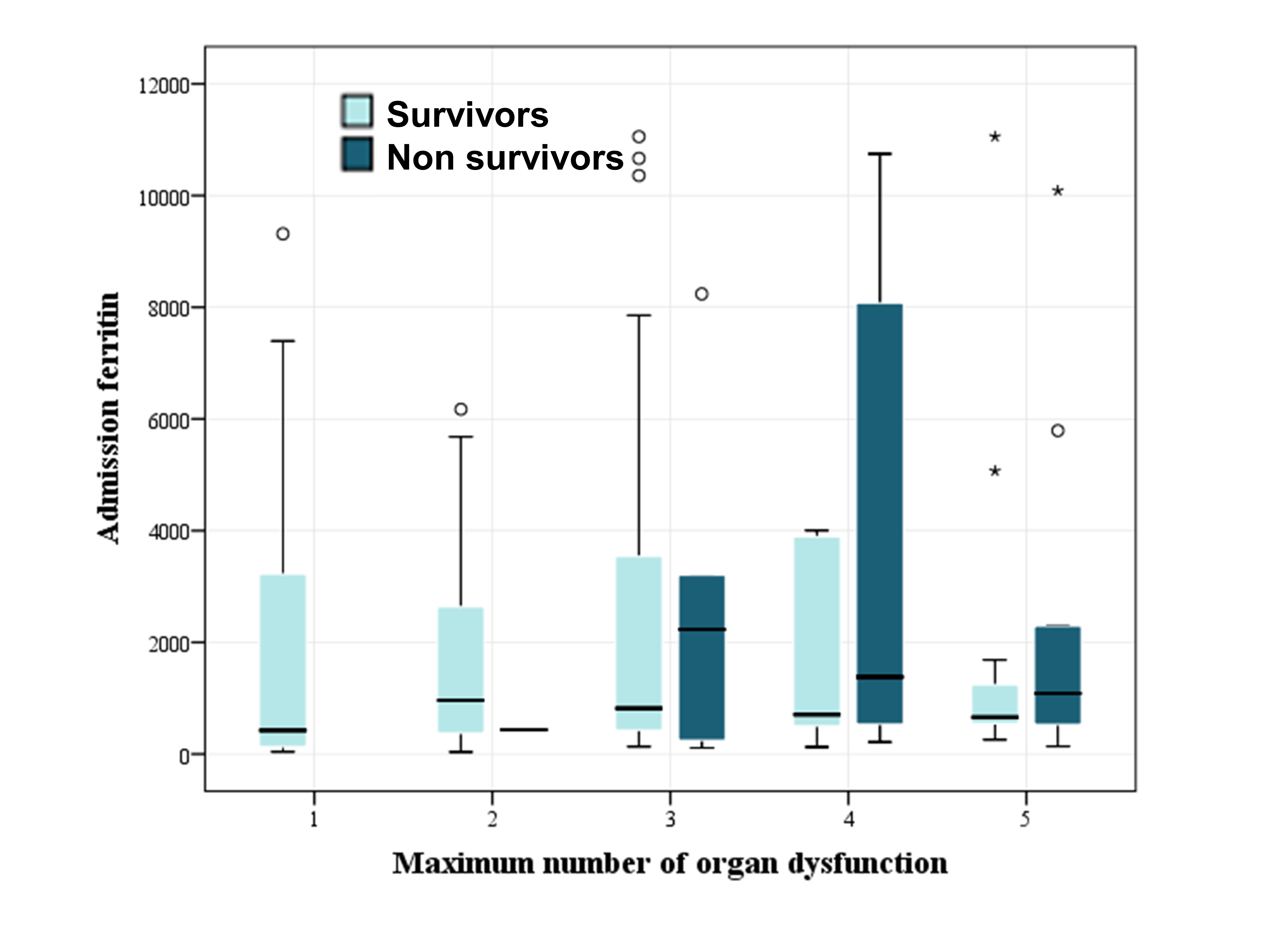

Supplement: Supplementary Figure 1 — Box plot comparing ferritin with degree of organ dysfunction and survival. [file Image_1.TIF]
