# Supplementary material for: Serum Ferritin Predicts Neither Organ Dysfunction Nor Mortality in Pediatric Sepsis Due to Tropical Infections
Source: Front Pediatr. 2020 Dec 3;8:607673. doi: 10.3389/fped.2020.607673 (PMC7747694; doi:10.3389/fped.2020.607673)
Supplement: Supplementary file 2 [file Table_1.docx]

**Supplementary Table 1. Predictive analysis of admission ferritin, percentage change of ferritin, PRISM III score, PELOD 2 score and MODS score for predicting 28 days mortality and NPMODS.**

| **Parameter** | **Value** | **Sensitivity** | **Specificity** | **AUC (95% CI)** | **P** |
| --- | --- | --- | --- | --- | --- |
| **Mortality** |  |  |  |  |  |
| Admission ferritin, µg/L | 500 | 62 | 36 | 0.53 (0.53, 0.67) | 0.73 |
|  | 3000 | 24 | 79 |  |  |
|  | 9700 | 14 | 91 |  |  |
| Percent decrease in ferritin | 25% | 67 | 31 | 0.50 (0.50,0.64) | 0.98 |
|  | 50% | 43 | 54 |  |  |
| PRISM III score | 10 | 81 | 67 | 0.82 (0.82,0.93) | 0.0001 |
| PELOD 2 score | 6 | 81 | 82 | 0.89 (0.89,0.95) | 0.0001 |
| MODS score | 3 | 95 | 60 | 0.83 (0.83,0.91) | 0.0001 |
| **NPMODS** |  |  |  |  |  |
| Admission ferritin, µg/L | 500 | 60 | 34 | 0.47 (0.39,0.61) | 0.82 |
|  | 3000 | 20 | 78 |  |  |
|  | 9700 | 10 | 99 |  |  |
| Percent decrease in ferritin | 25% | 60 | 27 | 0.42 (0.30,0.54) | 0.20 |
|  | 50% | 40 | 51 |  |  |
| PRISM III score | 10 | 70 | 68 | 0.73 (0.61,0.84) | 0.0001 |
| PELOD 2 score | 6 | 73 | 86 | 0.82 (0.71,0.94) | 0.0001 |
| MODS score | 3 | 83 | 61 | 0.77 (0.65,0.89) | 0.0001 |

PRISM - Pediatric Risk of Mortality, PELOD- Pediatric Logistic Organ Dysfunction, NPMODS- New or progressive Multiorgan dysfunction syndrome, AUC- Area under curve, CI- Confidence interval
