# Supplementary material for: Serum Ferritin Predicts Neither Organ Dysfunction Nor Mortality in Pediatric Sepsis Due to Tropical Infections
Source: Front Pediatr. 2020 Dec 3;8:607673. doi: 10.3389/fped.2020.607673 (PMC7747694; doi:10.3389/fped.2020.607673)
Supplement: Supplementary file 3 [file Table_2.docx]

**Supplementary Table 2. Sensitivity analysis comparing ferritin at admission and percentage change over 48 hours with mortality in those with definitive etiological diagnosis**

| **Parameter** | **Total**  **N=115** | **Survivors**  **N=99** | **Non survivors**  **N=16** | **P** |
| --- | --- | --- | --- | --- |
| Hyperferritinemia at admission, n (%) |  |  |  |  |
| Normal (≤300) | 19 (16.5) | 16 (16.2) | 3 (18.7) | 0.69 |
| Mild (301-1000) | 41 (35.7) | 37 (37.4) | 4 (25) |  |
| Moderate (1001-3000) | 29 (25.2) | 23 (23.2) | 6 (37.5) |  |
| Severe (3001-10000) | 13 (11.3) | 12 (12.1) | 1 (6.3) |  |
| Extreme (>10001) | 13 (11.3) | 11 (11.1) | 2 (12.5) |  |
| Median ferritin on day 1, µg/L | 791 (439,2876) | 698 (437,2876) | 1499 (573,2778) | 0.71 |
| Median ferritin on day 3, µg/L | 458 (249,820) | 459 (252,714) | 397 (173,2293) | 0.68 |
| Percentage change in ferritin | 48 (25,73) | 48 (25,14) | 41 (16,75) | 0.81 |
