# Supplementary material for: Serum Ferritin Predicts Neither Organ Dysfunction Nor Mortality in Pediatric Sepsis Due to Tropical Infections
Source: Front Pediatr. 2020 Dec 3;8:607673. doi: 10.3389/fped.2020.607673 (PMC7747694; doi:10.3389/fped.2020.607673)
Supplement: Supplementary file 4 [file Table_3.docx]

**Supplementary Table 3. Sensitivity analysis comparing ferritin at admission and percentage change over 48 hours with mortality in high risk organ dysfunction**

| **Parameter** | **Total**  **N=108** | **Survivors**  **N=71** | **Non survivors**  **N=37** | **P** |
| --- | --- | --- | --- | --- |
| Number of organ dysfunction, n(%) |  |  |  |  |
| 1 | 11 | 10 (14.1) | 1 (2.7) | 0.001 |
| 2 | 44 | 39 (54.9) | 5 (13.5) |  |
| 3 | 22 | 11 (15.5) | 11 (29.7) |  |
| 4 | 31 | 11 (15.5) | 20 (54.1) |  |
| Number of children with ferritin measurement at admission | **101** | **66** | **35** |  |
| Hyperferritinemia at admission, n(%) |  |  |  |  |
| Normal (≤300) | 15 (14.9) | 10 (15.2) | 5 (14.4) | 0.62 |
| Mild (301-1000) | 37 (36.6) | 26 (39.4) | 11 (31.4) |  |
| Moderate (1001-3000) | 22 (21.8) | 13 (19.7) | 9 (25.7) |  |
| Severe (3001-10000) | 10 (9.9) | 6 (9.1) | 4 (11.4) |  |
| Extreme (>10001) | 17 (16.8) | 11 (16.6) | 6 (17.1) |  |
| Median ferritin on day 1, µg/L | 811 (429,3546) | 755 (416,3392) | 1381 (434,5410) | 0.70 |
| Median ferritin on day 3, µg/L | 449 (234,954) | 453 (290,1001) | 254 (209,1030) | 0.28 |
| Percentage change in ferritin | 42 (19,69) | 41 (18,73) | 44 (21,67) | 0.89 |
