# Supplementary material for: Serum Ferritin Predicts Neither Organ Dysfunction Nor Mortality in Pediatric Sepsis Due to Tropical Infections
Source: Front Pediatr. 2020 Dec 3;8:607673. doi: 10.3389/fped.2020.607673 (PMC7747694; doi:10.3389/fped.2020.607673)
Supplement: Supplementary file 5 [file Data_Sheet_1.docx]

### Appendix 1. Definition of pediatric multiple organ dysfunction syndrome (MODS) as defined by Goldstein et al (21)

MODS is defined as the concurrent dysfunction of two or more systems. Each organ failure or dysfunction is defined by meeting one or more criteria of each organ or system.

**Cardiovascular dysfunction**

Despite administration of intravenous fluid bolus ≥40 mL/kg in 1 hour:

1) Decrease in blood pressure (hypotension) <5^th^ percentile for age or systolic blood pressure <2 SD below normal for age ^a^ **OR**

1. Need for vasoactive drug to maintain blood pressure in normal range (dopamine >5 μg/kg/min or dobutamine, epinephrine, or norepinephrine at any dose) **OR**
2. Two of the following
3. Unexplained metabolic acidosis: base deficit >5.0 mEq/L
4. Increased arterial lactate >2 times upper limit of normal
5. Oliguria: urine output <0.5 mL/kg/hr
6. Prolonged capillary refill: >5 seconds
7. Core to peripheral temperature gap >3°C

**Respiratory dysfunction ^b^**

1. PaO_2_/FiO_2_ <300 in absence of cyanotic heart disease or preexisting lung disease **OR**
2. PaCO_2_ >65 torr or 20 mm Hg over baseline PaCO_2_ **OR**
3. Proven need ^c^ for >50% FiO_2_ to maintain saturation ≥92% **OR**
4. Need for non-elective invasive or non-invasive mechanical ventilation ^d^

**Neurological dysfunction**

1. Glasgow coma score ≤11 **OR**
2. Acute change in mental status with a change in Glasgow coma score ≥3 points from abnormal baseline

**Haematologic dysfunction**

1. Platelet count <80,000/mm^3^ (<80 x 10^9^/L) or a decline of 50% in platelet count from highest value recorded over the past 3 days (for chronic haematology/oncology patients) **OR**
2. International normalized ratio (INR) >2

**Renal dysfunction**

Serum creatinine ≥2 times upper limit of normal for age or 2-fold increase in baseline creatinine

**Hepatic dysfunction**

1. Total bilirubin ≥4 mg/dL (not applicable to newborn) **OR**
2. Alanine transaminase (ALT) 2 times upper limit of normal for age

^a^Systolic blood pressure < 2 SD for age: 0 day – 1 week of age, < 59 mmHg; 1 week – 1 month, < 75; 1 month – 1 year, < 75; 2 – 5 years, < 74; 6 – 12 years, < 83; 13 – < 18 years, < 90 [5, 6].

^b^Acute respiratory distress syndrome (ARDS) must include a PaO_2_/FiO_2_ ratio ≤200 mm Hg, bilateral infiltrates, acute onset, and no evidence of left heart failure. Acute lung injury (ALI) is defined identically except the PaO_2_/FiO_2_ ratio must be ≤300 mm Hg.

^c^Proven need assumes O_2_ requirement was tested by decreasing flow with subsequent increase in flow if required.

^d^In postoperative patients, this requirement can be met if the patient has developed an acute inflammatory or infections process in the lungs that prevents him or her from being extubated.
